# Green Fluorescent Terbium (III) Complex Doped Silica Nanoparticles for TSPO Targeting

**DOI:** 10.3390/ijms20133139

**Published:** 2019-06-27

**Authors:** Elisabetta Fanizza, Nicoletta Depalo, Svetlana Fedorenko, Rosa Maria Iacobazzi, Alsu Mukhametshina, Rustem Zairov, Anastasio Salatino, Fabio Vischio, Annamaria Panniello, Valentino Laquintana, M. Lucia Curri, Asiya Mustafina, Nunzio Denora, Marinella Striccoli

**Affiliations:** 1 Dipartimento di Chimica, Università degli Studi di Bari Aldo Moro, Via Orabona 4, 70125-Bari, Italy; 2 CNR-Istituto per i Processi Chimico-Fisici SS Bari, Via Orabona 4, 70125-Bari, Italy; 3 Arbuzov Institute of Organic and Physical Chemistry, FRC Kazan Scientific Center of RAS, Kazan 420111, Russia; 4 IRCCS, Istituto Tumori “Giovanni Paolo II”-Viale Orazio Flacco, 65, 70124 Bari, Italy; 5 Dipartimento di Farmacia—Scienze del Farmaco, Università degli Studi di Bari Aldo Moro, Via Orabona 4, 70125-Bari, Italy

**Keywords:** TSPO ligand, nanoparticle-based fluorescent targeting, lanthanide complex probes

## Abstract

The low photostability of conventional organic dyes and the toxicity of cadmium-based luminescent quantum dots have prompted the development of novel probes for in vitro and in vivo labelling. Here, a new fluorescent lanthanide probe based on silica nanoparticles is fabricated and investigated for optically traceable in vitro translocator protein (TSPO) targeting. The targeting and detection of TSPO receptor, overexpressed in several pathological states, including neurodegenerative diseases and cancers, may provide valuable information for the early diagnosis and therapy of human disorders. Green fluorescent terbium(III)-calix[4]arene derivative complexes are encapsulated within silica nanoparticles and surface functionalized amine groups are conjugated with selective TSPO ligands based on a 2-phenylimidazo[1,2-a]pyridine acetamide structure containing derivatizable carboxylic groups. The photophysical properties of the terbium complex, promising for biological labelling, are demonstrated to be successfully conveyed to the realized nanoarchitectures. In addition, the high degree of biocompatibility, assessed by cell viability assay and the selectivity towards TSPO mitochondrial membrane receptors, proven by subcellular fractional studies, highlight targeting potential of this nanostructure for in vitro labelling of mitochondria.

## 1. Introduction

Organic [1,2,3,4,5] and organic/inorganic hybrid [6,7,8,9,10,11,12,13,14,15,16] nanoplatforms have been recently developed for biomedical applications, and have demonstrated to be able to play multiple functions that span from investigation of biological process, to imaging and drug delivery. Such nanoplatforms act as multipurpose scaffolds able to gain valuable information that cannot be easily achieved by using several single agents [15]. Smart multifunctional nanoparticles (NPs) can be prepared by combining contrast agents that may respond to different external stimuli (e.g., light, magnetic field, photoacoustic/thermoacoustic signal) and targeting ligands anchored at their surface, thus resulting in detectable nanosystems that can be selectively delivered at cellular or subcellular level, thanks to receptors recognition and/or binding to specific sites [14,16]. An accurate knowledge of the intracellular location of receptor is important for numerous areas of biomedical research, e.g., to understand the protein function and biological process [17]. Aberrant subcellular localization or levels of proteins have been reported to be related to the pathogenesis of many human diseases, such as metabolic, cardiovascular and neurodegenerative diseases, as well as cancer [18,19]. 

Translocator protein 18 kDa (TSPO), a five-transmembrane domains receptor mainly located in the outer mithochondrial membrane (OMM), has recently become an attractive subcellular target since it participates in a wide range of cellular functions, including cholesterol transport, steroid hormone synthesis, mitochondrial respiration, permeability transition pore opening, apoptosis, and cell proliferation [19,20]. Variations in TSPO expression have been also correlated to different diseases, from tumors to endocrine and neurological disorders [21]. Therefore, TSPO targeting is attractive for early detection of disease states that involve the overexpression of this protein and selective mitochondrial drug delivery [20]. Organic [2,3,20] and hybrid nanoplatforms [8,15] labelled with contrast agents and conjugated to TSPO ligands have been investigated as efficient in vitro TSPO targeting nanosystems. Although TSPO ligands that are active for positron emission tomography [22,23] and magnetic resonance imaging [15] have been developed, optical detection of TSPO targeting by means of fluorescence offers a versatile and prompt modality to monitor receptor localization. It is worth noting that, for subcellular targeting by means of exogenous (nanostructured) systems, cell interaction, internalization, selective ligand binding to the specific sites by molecular recognition and biodegradability [24,25,26,27] are crucial issues, being the most of these processes profoundly affected by size. Properly designed silica nanoparticles (SNs), characterized by a controllable size, good biocompatibility, biodegradability and stability in physiological conditions, and that can be easily functionalized represent promising platforms for biological application. Several colloidal synthetic strategies report on the formation of colloidal SNs hosting functionalities, including organic or inorganic probes [7,8,9,14]. Other post-preparative protocols describe that SNs surface can be promptly engineered, allowing chemically modify the SN surface to introduce reactive moieties, and provide functional groups to be further conjugated to the targeting ligand [16]. However, the size and surface area have been considered critical factors contributing to reducing the SNs biocompatibility, being cytotoxicity affected by the size-dependent cellular uptake. NP size, in fact, can induce cell damage, altering the cell function. NP size regulates their accumulation in cells and represents one of the critical parameters to be taken into account to design the suitable multifunctional nanostructures. In general, controversial results have been reported concerning the effect of SNs on cell viability, also demonstrating that different lines of the same cell type may respond differently to the same NP formulation. Yong Kim et al. [28] reported that, while considerable reduction of biocompatibility has been observed for SNs in the 100–200 nm size regime, probably due to cytotoxic effect induced by an energetically favored disruption of the cell membrane, SNs whose sizes fall in the range between 10 and 60 nm did not significantly affect the viability of several cell lines [28,29]. Size, together with shape, porosity for mesoporous silica-based nanostructures and in general surface area, also affect the degradation rate of the SNs being faster for smaller NPs rather than larger ones and slower for nonporous nanostructures compared to mesoporous ones [24,25,26,27]. 

Here, two samples of nonporous colloidal SNs, with average size of 22 nm and 46 nm, decorated with amino (NH_2_) groups and hosting a fluorescent probe, based on lanthanide complex, have been synthesized by means of a microemulsion approach, according to previously reported strategies [29,30,31,32], and further conjugated to a synthetic TSPO ligand based on 2-phenylimidazo[1,2-a]pyridine acetamide structure, containing derivatizable carboxylic end groups. Among the several type of ligands [20,21] used to target the TSPO receptor, the imidazopyridine acetamide based structures have been proved to selectively and specifically target the mitochondrial outer membrane TSPO protein [2,8]. A similar silica-based architecture using as fluorescent probe CdSe@ZnS quantum dots embedded in SNs has been previously synthesized by the authors and tested in TSPO targeting [8]. However, such a nanosystem [8] suffers from the inherent toxicity due to the presence of cadmium as demonstrated for in vitro traditional 2D cell culture [33,34], while higher cell viability values have been reported for quantum dots tested in 3D model cell [35]. Here, cadmium free SNs encapsulating lanthanide fluorescent probe based on Tb^III^ ions complex with p-sulfonatothicalix[4]arene (TCAS) ligand have been proposed as alternative optically traceable nanoplatforms for in vitro investigation on TSPO targeting. Fluorescence (PL) properties in lanthanide complex generally arise from an antenna mediated mechanism: the light absorbed by the antenna moiety is efficiently transferred to the lanthanide ions by energy transfer mechanism and fluorescent ion lines originate from the corresponding electron transitions [36]. Two emission mechanisms have been described: i. lanthanide ions, as Tb^III^ ions, emit a lower energy photon after absorption of a high-energy photon able to excite the molecular antenna, ii. lanthanide ions such as Er^3+^, Tm^3+^ and Ho^3+^, (with Yb^3+^ as sensitizer) demonstrated to have upconversion emission, they emit high energy photons in the ultraviolet/visible after absorbing two or more low-energy photons. According to the former mechanism, the photophysical properties of the Tb^III^ complex are characterized by narrow emission bands, large Stokes shift, larger than those of organic molecules, long lifetime and, moreover, they facilitate background elimination. The proposed Tb^III^-TCAS complex is based on a multicentered coordination of metal ions by means of calix[4]arene rims and it is characterized by efficient luminescence and good hydrophilicity both in neutral and weakly alkaline media [29,30,31,32]. However, in general, fluorescence properties of lanthanide complexes [36] undergo a significant deterioration in water due to quenching phenomena. In agreement with reported results [29,30,31,32,36,37,38], in general, a significantly improved photochemical stability can be achieved by taking advantage of the lanthanide complex encapsulation within a protecting silica shell, able to inhibit quenching phenomena due to diffusion of environmental ions or ligands and deterioration of the lanthanide-antenna ligands [32,36,37,38]. In addition, the functionalization of the SNs surface with NH_2_ groups allow them to be suitably conjugated to the TSPO ligands, as they expose a derivatizable carboxylic end group.

Photophysical and morphological characterization of this novel TSPO ligands functionalized lanthanide based SNs are reported here and the fluorescent nanoprobes cellular uptake and targeting of the TSPO receptor has been demonstrated, by means of subcellular fractionation experiments that have monitored the fluorescence of the separated cellular structure. The subcellular fractionation not only confirms the nanoprobes cellular uptake, but provides also a prompt quantitative evidence of the accumulation of the fluorescent probes in the subcellular compartments. In particular, being that the TSPO receptor is mainly located in the OMM, the higher PL intensity recorded from the mitochondria fractions confirms the potential of the TSPO ligand bound at the silica surface in selectively delivering the nanoprobes to the mitochondria, by means of receptor recognition.

## 2. Results.

### 2.1. Terbium Ion Doped Silica Nanoparticles

**Figure 1:**
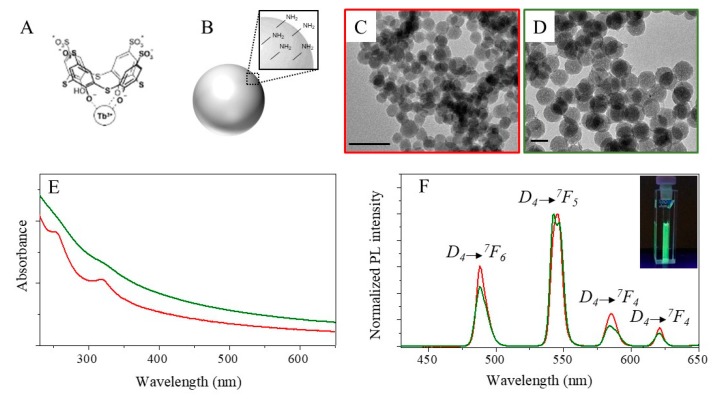
Sketches of Tb^III^-TCAS complex (**A**) and Tb^III^-TCAS complex doped silica nanoparticles functionalized with amine groups (**B**). TEM images (**C**,**D**, scar bar 50 nm), (**E**) UV-Vis absorbance and (**F**) fluorescent spectra at excitation wavelength of 330 nm of SN22 (**C**, red line in **E** and **F**) and SN46 (**D**, green line in **E** and F9) nanoparticles (NPs).

Green emitting SNs, doped with Tb^III^ complex with p-sulfonatothicalix[4]arene (Tb^III^-TCAS, Figure 1A) and surface functionalized by NH_2_ groups, have been prepared by a microemulsion approach (Figure 1B). Previous works [29,30,31] have extensively described the synthetic procedure of these lanthanide doped SNs, consisting in the dispersion of the aqueous solution of the luminescent Tb^III^-TCAS complex in cyclohexane and Triton X-100, followed by addition of aqueous ammonia solution and TEOS, which is the precursor of the silica matrix.

Two different samples of Tb^III^-TCAS doped SNs, referred to as SN22 and SN46, have been prepared by slightly changing the microemulsion mixture composition and synthetic procedure (see experimental section). TEM micrograph of the SN22 sample shows quite monodispersed (σ% of 12%) NPs with an average size of 22 nm (Figure 1C, see Figure S1A), while the SN46 sample is composed of NPs with an average size of 46 nm (σ% = 9%) (See Figure 1D and Figure S1B). In particular, the higher amount of TEOS used in the preparation of SN46 results in a shell thicker than that obtained for the SN22. It is worth noticing that the Tb^III^-TCAS complex, resulting from the coordination of Tb^III^ ions via three phenolate groups of TCAS^7-^ (schematically represented in Figure 1A), is not sufficiently water soluble to be entirely dissolved at the specific concentration (7.8 mM) which is used for the synthesis (for more details see Experimental Section).

Nevertheless, the complex added to the synthetic mixture in the form of aqueous colloids at pH 5–6 does not influence the shape and size of the NPs in the synthesis of large NPs as SN46, while the complex solubility is of key importance on size and shape uniformity of the smaller SN22. In previous reports [29,39] an improvement of the shape and size uniformity has been observed upon the addition of Tb^III^-TCAS in the form of true solution. The water solubility, in turn, has been reported to increase by the alkalization of the solution from pH ~6 to ~8 by pH-induced Tb^III^-(TCAS^7-^)-to-Tb^III^-(TCAS^8-^) transformation through the equilibrium (1)

Tb^III^-(TCAS^7-^) = Tb^III^-(TCAS^8-^) + H^+^ (1)

The surface decoration of both the SNs samples with NH_2_ groups was performed in the second step of the synthesis by adding APTES along with TEOS. The two-step addition of TEOS, in turn, is responsible for a gradient distribution of Tb^III^-TCAS within the silica matrix. In particular, the concentration of the Tb^III^-TCAS complex tends to decrease moving from core to surface of the NPs [31,40].

Decoration of the silica surface with NH_2_ groups allows accessing a versatile multifunctional platform, enabling covalent or coordination binding by means of the chemical reactivity of the functional groups. In addition, NH_2_ groups at the surface introduce a positive surface charge, conversely to the negative charge due to silanol groups on the bare silica surface. The ζ-potential for SN46 and SN22 have been found (+20.8 ± 0.3) mV and (+18.8 ± 0.6) mV, respectively. Quantification of NH_2_ groups for each sample has been carried out by an established colorimetric assay, based on the selective reaction of a ninhydrin solution with primary amine groups [41,42]. The SN46 has been found to have a concentration of 6·10^13^ NP/mL (calculated form the weight of the lyophilized sample 6.4mg/mL and assuming the density of the NP equal to that of pure silica nearly 2g/cm^3^) [30,41] and a concentration of NH_2_ groups of 5.0 mM that corresponds to number of amino groups per NP of 5·10^4^ (15 amine groups/nm^2^). Similar calculations allow to assess a SN22 sample concentration of 5·10^14^ NP/mL (weight of the lyophilized sample 4.2 mg/mL) and an NH_2_ groups concentration of 6.6 mM, corresponding to nearly 8000 amino groups per NP for the SN22 (13 amine groups/nm^2^).

**Figure 2:**
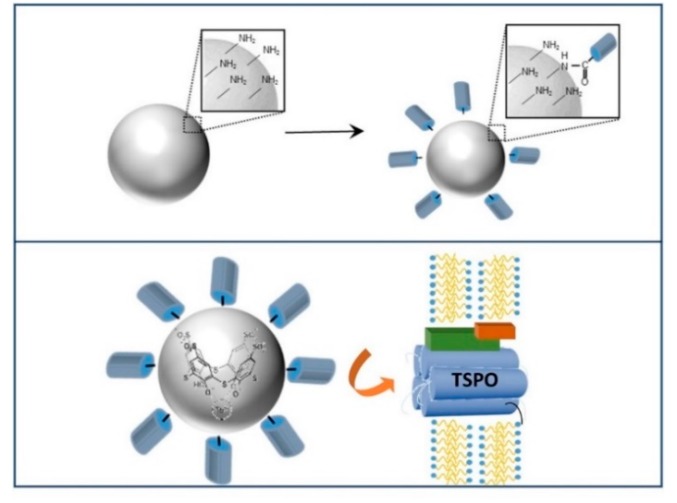
Sketches of the conjugation reaction of amino functionalized SNs with TSPO ligand (upper panel) and the translocator protein (TSPO) ligand functionalized silica NPs.

The spectroscopic characterization (Figure 1E,F) of the SN22 and SN46 confirms the efficient encapsulation of the lanthanide complex within the SNs. The Si: Tb molar ratios for the synthesized NPs calculated from the weights of Si and Tb quantitatively determined by ICP-OES measurements (Table S1) are 100:6 and 100:2 for SN46 and SN22 respectively. The smaller Tb content determined for SN22 versus SN46 correlates with the difference in phase and pH conditions applied in the synthesis of the NPs. In particular, the pH-induced deprotonation of the Tb^III^-TCAS complex in the synthetic conditions of SN22 can be accompanied by its partial degradation due to contribution of Tb^III^ hydrolysis. The UV-Vis absorbance line profile (Figure 1E) shows two faint bands at 245 nm and 316 nm, ascribable to the electronic transition of the Tb^III^-TCAS complex [29,30,31,32,39,43], superimposed to the low absorption and the high scattering of the silica matrix in the UV region, which is almost transparent in the visible range. Tb^III^-TCAS complex absorption features are very clearly visible in the absorption spectrum of the SN22, while only slightly perceptible in SN46, probably because in the latter case they are hidden by the large scattering contribution due to the thicker silica matrix. The PL spectra of the two samples (Figure 1F), excited at 330 nm (see Figure S2), clearly show the narrow emission bands characteristic of the terbium luminophore, arising from the transition from the excited D_4_ level to the four j levels (6, 5, 4, 3) of the ^7^F_j_, at 489 nm (D_4_ → ^7^F_6_), 541nm (D_4_ → ^7^F_5_), 582 nm (D_4_ → ^7^F_4_) and 620 nm (D_4_ → ^7^F_3_), respectively. It is worth noting the different spectral pattern of the Tb^III^- centered luminescence of SN46 and SN22 demonstrated by the different intensity ratio of D_4_ → ^7^F_5_ (541 nm) and D_4_ → ^7^F_6_ (489 nm) transitions. The SN46 sample presents an intensity ratio higher than that found for SN22. This result can be mainly ascribed to the terbium complex structure that, as previously described with Equation (1), depends on the pH-dependent acid-base equilibrium and is different for the SN46 and SN22 (Tb^III^-(TCAS^7-^) for SN46 prepared at Ph = 5–6 and Tb^III^-(TCAS^8-^) and SN22 prepared at pH = 8). Indeed, as reported in Figure S3 in the Supplementary Materials and highlighted in a previous work [44], pH-induced changes in the spectral pattern of the complex can be observed. In particular, the emission spectra highlight a general increase in the luminescence intensity moving to higher pH and a pH-dependent trend of the luminescence intensity ratio corresponding to the transitions at 541 and 489 nm. In particular, a decrease in the luminescence intensity ratio is observed moving from pH 5–6 to pH 8, thus confirming the lower intensity ratio measured for the SN22 with respect to SN46. Being that the emission at 541 nm is the most intense, both the samples show a green emission under UV-illumination (see picture in Figure 2F inset).

The absolute fluorescence quantum yields (PLQY) have been measured for the SN46 and SN22 samples, resulting in a value for SN22 (QY = 3) larger than that for SN46 (QY = 1.8). This trend agrees well with the above-mentioned structure modification of the Tb^III^-TCAS luminophores arisen from the difference in the specific synthetic step.

### 2.2. Functionalization of Green Fluorescent Terbium Ion Doped Silica Nanoparticles with Translocator Protein Ligand

An established procedure [8] has been here exploited to anchor a synthetic TSPO ligand, characterized by a 2-phenylimidazo[1,2-a]pyridine acetamide structure containing derivatizable carboxylic handles, at the SN surface, in order to fabricate luminescent nanostructures able to target TSPO protein (Figure 2).

The conjugation between the NH_2_ groups at the SN surface and the carboxylic moieties of the TSPO ligands is carried out in ethanol, as solvent, in the presence of BOP, as coupling agent, and DIPEA, an organic amine that regulates the pH, promotes the activation of the carboxylic moieties of the ligands and induces the fast aminolysis (Figure 2). Four hundred μL of each sample (that is 2 × 10^14^ NPs and 1.6 × 10^18^ total number of NH_2_ groups for SN22 and 2 × 10^13^ NPs and 1.2 × 10^17^ total number of NH_2_ groups for SN46) have been diluted to ethanol and a molar ratio between NH_2_ groups and TSPO ligand of nearly 2:1 has been used for the functionalization reaction in the presence of crosslinking agents, as reported in the experimental section. The large excess of NH_2_ groups is sufficient to ensure the conjugation of TSPO ligand, still avoiding the complete surface saturation. Indeed, the high binding ability demonstrated in previous studies [2,8] by the TSPO ligands suggested that the targeting moiety exposed on the surface of TSPO ligand-conjugated SNs can effectively recognize mitochondria even at low concentration [2,8]. In addition, the presence of residual free NH_2_ groups at the surface provides a positively charged surface, which promotes the TSPO-targeted SNs internalization and subsequent delivery to mitochondria. Indeed, upon conjugation, the ζ-potential value drops down to (+10.4 ± 0.6) mV for the TSPO ligand-conjugated SN22 (from +18.8 mV of the as prepared SN22) and to (+16.4 ± 0.5) mV for the TSPO ligand-conjugated SN46 (from +20.8 mV of the SN46), being still positive, and thus confirming the presence of residual primary NH_2_ groups on SN surface.

**Figure 3:**
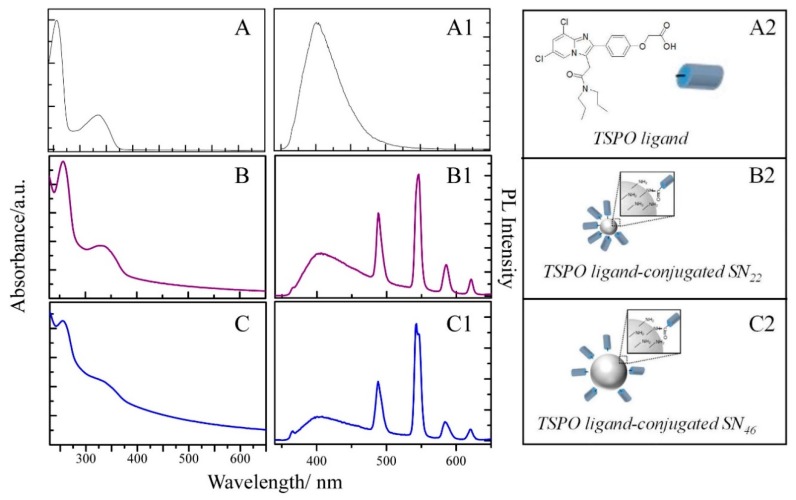
UV-Vis absorbance (**A**,**B**,**C**) and emission (A1,B1,C1) spectra of the TSPO ligand (A,A1, molecular sketch in panel A2), TSPO ligand-conjugated SN22 (B,B1, sketch of the multifunctional NP in panel B2) and SN46 (C,C1, sketch of the multifunctional NP in panel C2).

**Figure 4:**
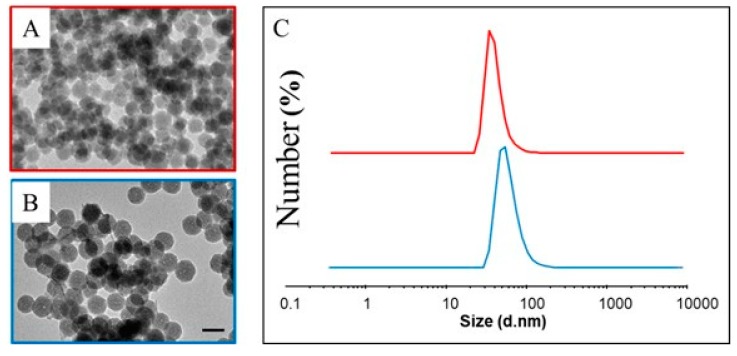
TEM micrographs and size distribution measured by DLS analysis of the TSPO ligand conjugated SN22 (**A**, **C** violet line) and SN46 (**B** and **C** blue line).

The UV-Vis absorbance spectra of the TSPO ligand conjugated SN22 (Figure 3B) and SN46 (Figure 3C), show both the characteristic bands at 330 nm and 227 nm of the TSPO ligand (Figure 3A) superimposed on the profile characteristic of the silica matrix. The absorption features of the TSPO ligand completely hide those of the Tb^III^-TCAS complex. However, the emission spectra for the two samples (Figure 3B1,C1) confirm the presence of the lanthanide complexes in the silica matrix, as indicated by the characteristic multiple bands structure of the Tb^III^-TCAS complex. See Figure 1F, recorded exciting at 330 nm, together with the broad emission band of the TSPO ligand (Figure S1A1) at 401 nm. Blank experiments carried out by mixing the SN with the TSPO ligand without crosslinking agents allows to rule out non-specific ligand adsorption onto the silica surface under these experimental conditions. Finally, the spectroscopic characterization reported in Figure 3 confirms the success of the functionalization of the Tb^III^-TCAS doped silica matrix with TSPO ligand bound at its surface. After functionalization, SNs preserve their size, as revealed by the morphological characterization in Figure 4A,B (See Figure S1A1,B1) and by DLS analysis of the two samples.

DLS investigation shows that bio-conjugates are characterized by a monomodal size distribution, with average diameters of about 36 nm (PDI 0.151 ± 0.015) and 58 nm (PDI 0.168 ± 0.016) for TSPO ligand functionalized SN22 (Figure 4C, violet line) and SN46 NPs (Figure 4C, blue line), respectively.

A calibration curve, reporting the emission intensity at the emission maximum of TSPO ligand (401 nm) versus the ligand concentration (See T4), allows estimating the amount of TSPO ligand bound at the silica surface. It is worth noting that, at this emission wavelength (401 nm), the PL is only due to TSPO ligand. A concentration of 6 × 10^−5^ M (0.03 mg/mL) of TSPO ligand for TSPO ligand-conjugated SN46 (8.4 mg/mL) and 2 × 10^−4^ M (0.9 mg/mL) for SN22 (7.1 mg/mL) is measured.

### 2.3. In Vitro Cytotoxicity and Intracellular Fate of TSPO Ligand-Conjugated SNs

**Figure 5:**
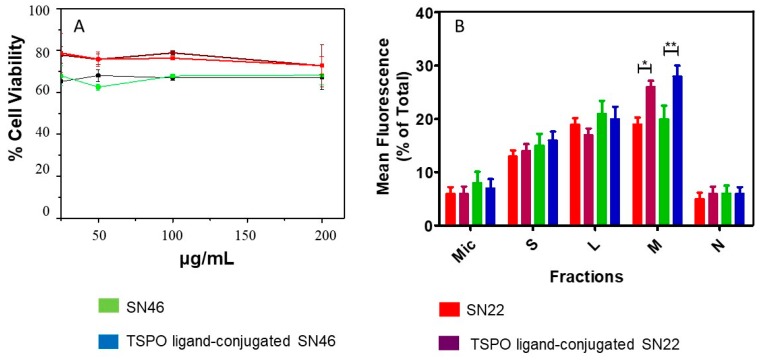
Cell viability profiles of U87MG cells incubated with amino functionalized SN22 and SN46 samples before (**A**, red line and green line, respectively) and after (**A**, wine line and blue line, respectively) their conjugation with TSPO ligand, for 24 h. Each point represents a mean value of three independent experiments. Subcellular fractionation of U87MG cells after incubation with amino functionalized SN22 and SN46 NPs (**B**, red and green bars) and TSPO ligand bioconjugates (**B**, vine and blue bars). Key: microsomal (Mic), soluble (S), lysosomal (L), mitochondrial (M) and nuclear (N) fractions. Statistical values *p* < 0.05 (*) and *p* < 0.01 (**) estimated by two-way ANOVA and Bonferroni post hoc test. Data represents mean ± SD, *n* = 3.

In vitro cytotoxicity experiments and intracellular fate study have been carried out by incubating the SN22 and SN46 samples before and after TSPO ligand conjugation with U87-MG human glioblastoma cancer cells, purposely chosen as in vitro cancer model since they overexpress the TSPO receptor [45,46]. The effect of the luminescent silica-based nanostructures on cell viability has been evaluated by using the conventional MTT assay, after incubation of U87MG cells with the amino functionalized samples, namely SN22 and SN46, and the two TSPO targeted samples, for 24 h (Figure 5). The doses of the SN22 and SN46 and TSPO ligand conjugated SN22 and SN46 samples have been varied in the range between 25 and 200 μg/mL. U87MG cells have shown a dose independent reduction in viability, in the tested concentration range, after 24 h of exposure of SN22 and SN46 samples, before and after their conjugation with TSPO ligand. In addition, a size dependent effect on cell viability has been observed. The large SN46 sample has resulted in a cell viability slightly more reduced than that found for the small SN22 samples. Anyhow, a percentage of cell viability higher than 50% has always been recorded, in the tested concentration range.

Subcellular fractionation experiments have been performed on U87MG cells for a quantitative evaluation of the possible targeting ability of the prepared TSPO ligand conjugated SNs at subcellular level. Namely U87MG cells have been incubated with the two different size samples, both targeted and on targeted SN (0.05 mg/mL) for 12 h and nuclear (N), mitochondrial (M), lysosomal (L), microsomal (Mic) and soluble (S) cell fractions [8] were separated by differential centrifugation.

The emission band at 545 nm (excitation wavelength at 330 nm), characteristic of the Tb^III^-TCAS complex doped SNs, has been used to evaluate the amount of silica-based nanostructures accumulated in the different cell compartments.

The comparison of the monitored mean PL intensity, expressed as percentage of the total PL, for lysosomal (L), nuclear (N), microsomal (Mic), soluble (S) and mitochondria (M) fractions after incubation of U87MG cells with bare and targeted SNs, respectively, for both the samples (SN22 and SN46), is reported in Figure 5. A higher PL intensity has been detected in mitochondrial and lysosomal fraction, although a not negligible mean PL intensity has been recorded in the soluble fraction, while less intense mean PL values have been found in the nuclear and microsomal fractions, for the all investigated targeted and no targeted samples. Interestingly, a statistically significant difference in the mean PL intensity has been observed only by comparing PL values, recorded in the mitochondrial fraction, of amine functionalized SNs and the corresponding TSPO ligand bioconjugates, for the two size different samples. Namely, for SN22 samples, a mean fluorescence value of about 26% and 18% for TSPO targeted and no targeted NPs, respectively (*p* < 0.05) has been obtained from the mitochondrial fraction. In the case of SN46 samples, the mean PL value has been found 19% and 29% for amino functionalized and targeted luminescent NPs, respectively (*p* < 0.01).

## 3. Discussion

In general, a fluorescent biological label needs to be biocompatible, dispersible in biological medium, easily functionalized with specific organic or bio-organic groups suitable for targeting specific receptor sites [47,48] and, in addition, its emission properties should be suitable for in vitro or in vivo optical labelling and imaging [49]. The reported fluorescent lanthanide nanosystem based on Tb^III^-TCAS complex doped silica NPs decorated with NH_2_ groups and functionalized with TSPO ligand, meets most of these requirements.

Several papers have already described the antenna mechanism behind the improved PL in Tb^III^-TCAS complex [44]. TCAS represents one of the possible antenna structures that enables obtaining very stable terbium ions complex through a multicentered coordination of the Tb^III^ ions, thus facilitating the luminescence properties through a ligand-to-metal energy transfer, and the sulfonate groups make the Tb^III^-TCAS rather hydrophilic at pH>8 [31,50]. Activation of the antenna mechanism using TCAS occurs in the excitation range between 300 and 400 nm where the phenolate sulfonate rings of the TCAS can absorb light inducing Tb^3+^ sensitization by an energetically favored singlet-triplet intersystem crossing transition (in the TCAS antenna), followed by energy transfer from the TCAS to the terbium centre [51]. Additional advantages are gained by encapsulating the Tb^III^-TCAS complex inside the silica matrix [29,30,31,32]. The hydrophilicity of the silica shell balances the poor solubility in water of the Tb^III^-TCAS complex. Meanwhile, trapping the lanthanide complex within the silica matrix reduces the environmental induced quenching, taking advantage of the protection provided by the silica matrix to the Tb^III^-TCAS. However, a slightly smaller PLQY is measured for the SN46 sample compared to the SN22, due to both the pH-induced structure modification of the Tb^III^-TCAS luminophore arisen from the above-mentioned difference in the specific synthetic step and a thicker silica shell in the former sample that reduces the excitation due to scattering phenomena. Further, the versatile silica surface, easily decorated with NH_2_ groups by condensation of APTES molecules with the silanol groups, offers a suitable platform for subsequent conjugation with TSPO ligands, able to induce specific subcellular targeting. Even though the two samples have a different concentration in terms of number of NPs for mL and number of NH_2_ groups per NP, the NH_2_ groups density (amine groups/nm^2^) is almost the same (13 amine groups/nm^2^ for the SN22 and 15 amine groups/nm^2^ for the SN46). The higher content of TSPO ligand bound at the SN22 surface, qualitatively demonstrated from UV-Vis absorbance profiles and quantitatively measured from fluorescence spectra, can be ascribed to the higher NP concentration of SN22 sample used for the conjugation reaction. In addition, the decrease in NP size results in a concomitant increase in NP curvature and, due to a lower packing of the surface functional groups, in a reduced surface steric hindrance, therefore TSPO ligands conjugate at the surface amine groups more easily.

Irrespectively from the size and the surface chemistry, that is mainly based on NH_2_ and silanol groups for SN22 and SN46, TSPO ligand and residual NH_2_ and silanol groups, for the TSPO ligand conjugated samples, the terbium doped silica based nanosystems can be easily dispersed in an aqueous medium, as confirmed by ζ-potential measurements. In vitro cytotoxicity study performed on U87-MG human glioblastoma cancer cells, that overexpress protein TSPO [45], and assessed by MTT method, demonstrates that cell viability is only partially affected by exposure to amine functionalized SNs and the corresponding TSPO ligand bioconjugates, for the two different size samples. Indeed, for both the amino functionalized and TSPO-conjugated SNs, a cell viability percentage of 65% for the SN46 and 73% for the SN22 at the highest tested dose (200 μg/mL), respectively, is obtained, thus indicating a good degree of biocompatibility of the luminescent silica based nanostructures, in the tested experimental conditions. Anyhow, a dose-independent, but size-dependent reduction in cell viability is achieved in the investigated concentration range, at 24 h of cell incubation with amine or TSPO functionalized SN22 and SN46 samples. In particular, the larger SN46 sample, (about 46 nm by TEM investigation) have mostly affected the viability of U87-MG cells when compared to the SN22 nanostructures (about 20 nm by TEM analysis), irrespectively from the surface chemistry, i.e.,; with or without TSPO ligand on their surface. Size-dependent cytotoxic effects have been already observed, for silica-based NPs, in several reports, highlighting the occurrence of a strong correlation between the specific toxicity response and the tested experimental conditions, as cell type, cell line, incubation time and cytotoxicity assay method [28]. In-Yong Kim et al. [28] proved that several complex mechanisms, such as reactive oxygen species (ROS) formation and deformational energy changes in membrane integrity and fluidity, involved in endocytic and non-endocytic cellular uptake, concur to affect the cell viability induced by cell exposition to silica NPs with different size. Larger silica NPs (60 nm) resulted more toxic than smaller ones (20 nm), at the specific tested concentration values and for the selected cell lines, due to a more significant membrane cell injury and cellular induced-ROS damage [28]. This trend supports our findings relative to the observed effects induced on cell viability by the two different size tested SNs samples, with or without TSPO ligand conjugated to their surface.

The potential of the designed luminescent silica based nanostructures to recognize the TSPO receptor and to localize in the mitochondria subcellular compartment is, here, proved by subcellular fractionation and PL detection of the characteristic Tb^III^-TCAS fluorescence (excitation wavelength 330 nm) measured from each cellular fraction (nuclear (N), mitochondrial (M), lysosomal (L), microsomal (MIC) and soluble (S)). Subcellular fractionation and PL detection provides qualitative and quantitative results, corroborating the intracellular fate, answering the urge of more quantitative methods able to define intracellular compartmentation. The subcellular fraction study demonstrates that both TSPO targeted and not targeted NH_2_-grafted SNs, with the two different sizes, are able to cross the extracellular membrane and to be internalized in the cells by both endocytic and non-endocytic pathways [8,28]. Anyhow, the PL emission intensity values observed for the lysosomal fraction of both two different size SNs, targeted and not targeted, seem to suggest that their cellular internalization occurs by means of endocytic pathways that enable NPs accumulation in the endosomal/lysosomal compartments of cells. A significant fraction of the positively charged NPs is also able to disrupt the endosomal organelles reaching the cytosolic space in living cells [8], as mainly indicated by PL intensity values of the soluble fraction. Furthermore, the NP localization in subcellular compartments different from lysosomes demonstrated the SNs ability, not only to achieve the endosomal/lysosomal escape, but also to be cell internalized, concomitantly, by means of non-endocytic pathways (or passive transport) [28]. Interestingly, the PL intensity values recorded in the mitochondrial fraction, after cell exposure to TSPO targeted SNs, for the two differently sized samples, demonstrate the good selectivity of the luminescent TSPO functionalized nanostructures to perform the mitochondrial targeting. Conversely, no statistically significant difference has been observed between the PL intensity values of lysosomal and soluble fraction for the not targeted SN22 and SN46 samples. These results demonstrate that the TSPO ligands anchored to SNs surface are able to recognize to a significant extent the specific mitochondrial membrane receptor, thus providing luminescent silica-based nanostructures that can be delivered and revealed, at subcellular level, in the mitochondria.

## 4. Materials and Methods

### 4.1. Materials.

Tetrathyls orthosilicate (TEOS, 98%, ammonium hydroxide (28–30%), terbium(III) nitrate hexahydrate (99.9%) cyclohexane were purchased by Acros; 2,6-lutidine (98%, Alfa Aesar, Kandel, Germany), ninhydrin, Triton X-100, 3-aminopropylstriethoxysilane (APTES), benzotriazol-1-yloxy)tris-(dimethylamino)phosphonium hexafluorophosphate (97%, BOP) and N,N-diisopropylethylamine (99.5% DIPEA) were purchased from Sigma-Aldrich (Milan, Italy). All chemicals, purchased from Sigma Aldrich if not specified otherwise, were used without purification. The TSPO ligand 2-(6,8-dichloro-2-(4-hydroxyphenyl)imidazo[1,2-a]pyridin-3-yl)-N,N-dipropylacetamide (MW = 477 g·mol^−1^) was prepared according to synthetic procedures reported elsewhere [2]. The synthesis of p-sulonatocalix[4]arene tretasodium (TCAS) salt was carried out according to the classical procedure [52].

Human glioblastoma cancer cells, U87-MG, were grown in DMEM high glucose medium supplemented with 10% heat-inactivated fetal bovine serum (FBS), 100 U/mL penicillin, 100 μg/mL streptomycin and 2 mM L-glutamine in a 5% CO_2_ humidified atmosphere at 37 °C. All materials for cell culturing were purchased from EuroClone, Italy. Disposable culture flasks and Petri dishes were from Corning, Glassworks (Corning, NY, USA). 3-(4,5-Dimethylthiazolyl-2)-2,5-diphenyltetrazolium bromide (MTT) was purchased from Sigma-Aldrich (Milan, Italy).

### 4.2. Synthesis of Terbium Doped Silica Nanoparticles

Synthesis of 22 nm sized amino modified Tb^III^-doped silica nanoparticles (SN22) was performed by mixing Triton X-100 (1.25 g), cyclohexane (20 mL), TEOS (0.16 mL) with 0.18 mL of the aqueous solution of [Tb(TCAS)] complex [29,30,31,32] (C_[Tb(TCAS)]_ 7.8 mM, pH = 8, with NaOH). The mixture was stirred for 20 min and a following addition of 0.12 mL of aqueous ammonia (28–30%) was performed. Then, the mixture was stirred for 24 h before adding TEOS (0.08 mL) and, 30 min later, APTES (0.022 mL), keeping than further stirring for 24 h. The obtained NPs were collected by adding acetone and washed with acetone/ethanol mixture (1:1) and water through two centrifugation/redispersion steps. The synthesis of Tb-doped SNs of 46 nm in size, covalently decorated by amino groups (SN46), was performed with the use of APTES in accordance with the established procedure. An aqueous suspension of luminescent complex Tb-TCAS (C=7.8 mM, 2.16 mL, without adding of NaOH, Ph = 3–4) was added dropwise to a mixture of Triton X-100 (8.63 g), cyclohexane (33.75 mL), and *n*-heptanol (8.1 mL) with stirring for 15 min. Then aqueous ammonia (30 %) was added (0.27 mL) to the mixture. After stirring for 15 min, TEOS (0.23 mL) was added and the reaction was continued for 24 h. Then TEOS (0.23 mL) was added to the mixture, followed by the addition of APTES (0.045 mL) 30 min after. The reaction continued for another 24 h. The obtained NPs were collected by adding acetone and washed with acetone/ethanol mixture (1:1) and water through two centrifugation/redispersion steps. Concentration of amine groups on SNs surface was performed by using a ninhydrin test [8,41,42]. The ninhydrin solution was prepared by dissolving 110 mg of ninhydrin in 16 mL of ethanol (0.68% *w/v*) and 4 mL of 2,6 lutidine. 950 µL of the ninhydrin solution was added to 50 µL of SN46 and SN22, warmed up at 80 °C for 10 min. The suspension turns light blue due to the formation of the Ruhemann’s Blue by-product, highlighting the presence of amine groups. The UV-Vis absorbance spectrum shows the occurrence of a band centered at 570 nm characteristic of the Ruhemann’s Blue formation. A calibration curve obtained by carrying out the ninhydrin assay on a set of stock solutions of APTES at different concentration allows to estimate the extinction coefficient (Ɛ = 2678 ± 117 L·mol^−1^ ·cm^−1^), and hence to establish the concentration of primary amine groups from the Lambert-Beer law. Samples were lyophilized in order to determine their weight.

### 4.3. Conjugation Reaction of the TSPO Ligand onto SN22 and SN46 Samples

Conjugation reaction was carried out by mixing suitable amount of NPs suspension (400 µL) and TSPO ligand in ethanol (1.5 mL) in the presence of BOP and DIPEA, as cross-coupling agents. The reagents were mixed using amine groups: TSPO ligand molar ratio 2:1 DIPEA:TSPO ligand 3:1 and BOP:TSPO ligand 1.2:1 molar ratio, respectively. The suspension was stirred at room temperature for 48 h and the TSPO-functionalized NPs were collected by centrifugation at 7800 g for 10 min (Beckman J2-21), purified by three cycles of re-dispersion in ethanol/centrifugation and dispersed in 1 mL of ethanol for further spectroscopic characterization. The TSPO content in TSPO conjugated SNs was determined by first creating of a calibration curve by plotting the emission intensity at 400 nm (excitation wavelength 330 nm) versus the TSPO ligand concentration for a set of TSPO ligand solutions in the range of 10^−5^–10^−7^ M (Ɛ_254 nm_ = 35945 ± 710 L mol^−1^·cm^−1^). Finally, once the extinction coefficient was known, the content of TSPO conjugated onto TSPO ligand conjugated SNs was calculated.

### 4.4. Characterization

UV-Vis absorbance spectra were recorded by a Cary Varian 5000 UV-visible NIR spectrophotometer and photoluminescence spectra were recorded using a spectrofluorimeter Fluorolog 3 (HORIBA Jobin-Yvon) equipped with double chromator reticles in excitation and emission. All optical measurements were carried out at room temperature. Absolute PL quantum yield was measured by means of a “Quanta-phi” integrating sphere coated with Spectralons^®^ and mounted in the optical path of the spectrofluorometer, using as an excitation source a 450 W xenon lamp coupled with a double-grating monochromator. Transmission electron microscopy (TEM) analysis has been performed by using a JEOL 100, operating at 100 kV and equipped with a W electron source and a CCD high resolution camera. Deposition of the NPs was carried out by dipping a carbon coated copper grid in the SNs solution and let the solvent to evaporate. Size statistical analysis (average NPs size and relative standard deviation (σ%)) for each NP.

A Zetasizer Nano ZS, Malvern Instruments Ltd., Worcestershire, UK (DTS 5.00) was used to determine hydrodynamic diameter (size), size distribution and colloidal stability of the SNs, before and after conjugation with TSPO ligand. In particular, aqueous solutions (PBS buffer, 10 mM, pH 7.2) of SNs were diluted in demineralized water and sonicated for 3 h to measure size and size distribution by means of dynamic light scattering (DLS). Polydispersity index (PDI) was employed to describe the SNs size distribution and hydrodynamic diameters were reported by number. A laser Doppler velocimetry (LDV) used to perform the ζ-potential measurements after dilution of SNs aqueous solution (PBS buffer, 10 mM, pH 7.2) in KCl aqueous solution (1 mM) and sonication for 3 h. All reported data represent average values (± standard deviation) of three replicates.

Content of Tb and Si in the synthesized colloids was measured using inductively coupled plasma optical emission spectrometry (ICP-OES) model iCAP 6300 DUO by Varian Thermo Scientific Company equipped with a CID detector. This spectrometer enables the simultaneous measurement of peak heights within the 166 to 867 nm range. The optical resolution is less than 0.007 nm to 200 nm. The working frequency is 27.12 MHz. Together, the radial and axial view configurations enable optimal peak height measurements with suppressed spectral noises. The experimentally observed Tb (spectral line—50.917 nm) and Si (spectral line—251.611 nm) concentrations are summarized in Table S1 in the Supplementary Materials.

### 4.5. Cytotoxicity Assays and Intracellular Fate of SN22 and SN46 before and after TSPO Ligand Conjugation

U87-MG human glioblastoma cancer cells were seeded in 96 wells plates at a density of 5000 cells/well. After 24 h, the culture medium was replaced with dilution in fresh medium (100 µL) of compounds in the range 0.625–200 µg/mL, in order to evaluate both the proper concentration for uptake experiments and the antiproliferative effects on cell viability of compounds. MTT assay was performed as previously described [53] in order to study the cytotoxicity of compounds. Results were expressed as percentage of cell viability at the highest tested dose. For the SNs intracellular fate study, cell fractionation of U87 MG cells was performed by differential centrifugation. In particular, U87 cells were incubated for 12 h with TPSO targeted and not targeted SNs at a concentration of 0.05 mg/mL. Subsequently the extracellular content was washed off with cold PBS and then the pellet was washed once with the homogenization buffer (250 mM sucrose, 10 mM HEPES, 1 mM EDTA, pH 7.4) and the cocktail aprotinin protease containing (2 μg/mL), leupeptin (2 μg/mL), pepstatin A (1 mg/mL) and PMSF (1 mM). Then, differential centrifugation was conducted and the different cellular fractions of U87 MG cells: nuclear (N), mitochondrial (M), lysosomal (L), microsomal (MIC) and soluble (S) were collected. Each fraction was diluted with PBS and the fluorescence intensity at 545 nm (excitation wavelength at 330 nm) was measured in a microplate reader TECAN (Application: Tecan i-control, Device: infinite M1000Pro). Statistical values *p* < 0.05 (*) and *p* < 0.01 (**) were estimated by using two-way ANOVA and Bonferroni post hoc test. Data represents mean ± SD, *n* = 3

## 5. Conclusions

Two different samples of cadmium free SNs encapsulating lanthanide fluorescent probe based on Tb^III^ ions complex with p-sulfonatothicalix[4]arene (TCAS) ligand have been synthesized and conjugated with the synthetic TSPO ligand. The encapsulation of the Tb^III^-TCAS complex into SNs has enabled their stabilization in aqueous medium concomitantly preserving its peculiar optical properties from quenching phenomena. Indeed, the resulting luminescent nanostructures are characterized by good colloidal stability in physiological media and a final average hydrodynamic diameter, provided by DLS analysis, of 36 and 58 nm, for the two different samples, respectively. The cytotoxicity study has proven that the cell viability of U87-MG human glioblastoma cancer cells has been only minimally affected upon their incubation for 24 h with the Tb^III^-TCAS doped SNs in the explored concentrations range, up to 200 µg/mL. The results of the subcellular fractionation experiment have clearly demonstrated the occurrence of the cellular uptake of both targeted and not targeted SNs, with a statistically significant accumulation of targeted SNs in the mitochondria with respect to their not targeted counterparts. Therefore, the obtained luminescent silica nanostructures hold a good potential for future applications in diagnostics and therapeutics. Indeed, these nanostructures present an enhanced degree of biocompatibility and a significant ability to recognize the specific mitochondrial membrane receptor, thus resulting promising optically traceable candidates that can be used as an alternative to cadmium-based nanoparticles for the future specific targeting studies on imaging of TSPO protein. The increase in the mean fluorescence value recorded in the mitochondrial fraction for the targeted Tb^III^-TCAS doped nanoparticles with respect to the non-targeted counterpart, when tested in U87-MG cells, is lower than that recorded in the targeted and not targeted quantum dots based SNs when tested in C6 glioma rats cells, however, such an increase in fluorescence intensity stays statistically significant for both the two Tb^III^-TCAS doped nanoparticles samples with the different sizes (*p* < 0.05 and *p* < 0.01). The difference in the targeting ability observed for Tb^III^-TCAS doped and quantum dots based SNs could possibly arise from different tested cell lines. In fact, since the nature of luminescent probe embedded in the SNs presumably does not affect the TSPO targeting ability of TSPO ligand located on the surface of SNs, and being that size and surface chemistry are comparable for both the Tb^III^-TCAS doped and the quantum dots containing silica nanostructures, the same cellular fate and, accordingly, the same recognition mechanism, can be reasonably assumed in the two cases.

The high versatility of the proposed procedure for the fabrication of the Tb^III^ complex doped silica-based nanoparticles enables, in principle, its implementation for another lanthanide doped SNs. Remarkably, the modification of the chemical composition of the fluorophore inside the SNs is realistically not expected to affect the ligand mediated target ability, as it results from the silica surface chemistry and the recognition interaction of the bioconjugated ligand. On this basis, exploration of the encapsulation route for alternative fluorofores, such as upconverting lanthanide ions, which are particularly amenable for biomedical applications, such as labelling, detection, imaging and therapy due to sharp emission peaks, absence of autofluorescence photobleaching and chemical degradation under near infrared radiation, high signal-to-background ratio, long luminescence lifetime and large Stokes-shift. Further development of the study will be devoted to exploration of cell lines with different TSPO expression level to evaluate the in vitro cytotoxicity of the developed lanthanide doped SNs, and to assess their TSPO targeting ability, and hence labelling potential, by means of kinetic/subcellular fraction experiments and confocal microscopy analysis.

## Supplementary Materials

Supplementary materials can be found at https://www.mdpi.com/1422-0067/20/13/3139/s1.
