# Supplementary material for: Green Fluorescent Terbium (III) Complex Doped Silica Nanoparticles for TSPO Targeting
Source: Int J Mol Sci. 2019 Jun 27;20(13):3139. doi: 10.3390/ijms20133139 (PMC6651519; doi:10.3390/ijms20133139)
Supplement: Supplementary file 1 [file ijms-20-03139-s001.pdf]

Supplementary materials

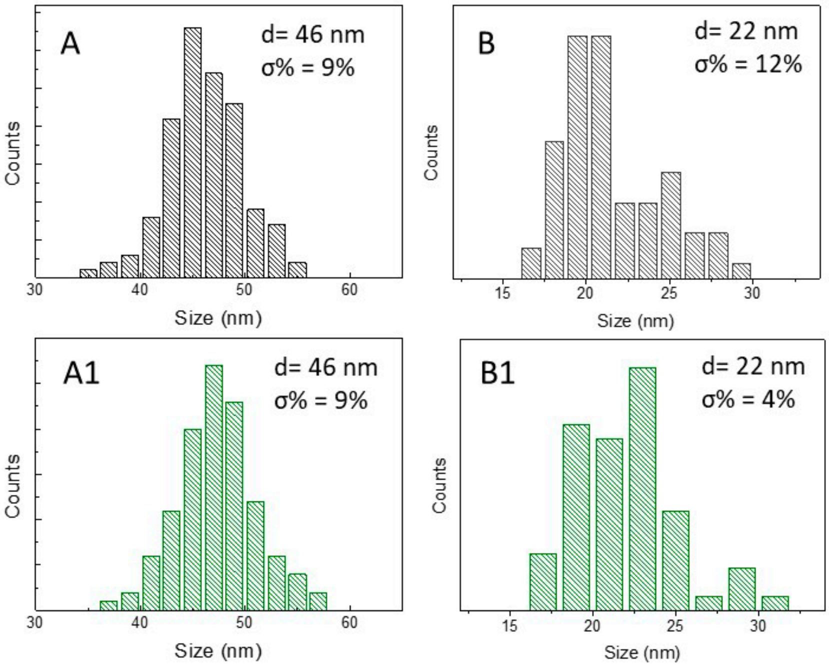

Figure S1. Statistical analysis of the size of the Tb^{III}-TCAS doped SN NP samples measured form TEM micrographs. Amino functionalized silica sample SN46 (**A**), SN22 (B) and TSPO ligand conjugated SN 46 (A1) and SN22 (B1).

Table S1. ICP-OES data of the synthesized Tb(III) doped silica nanoparticles (C_{SNs}=0.2 g L^{-1}).

**Sample**	**Element content mg L^{-1}, ±10%**	
	**Si (251.611 nm)**	**Tb (350.917 nm)**
SN22	89.5	1.18
SN46	75.5	3.22

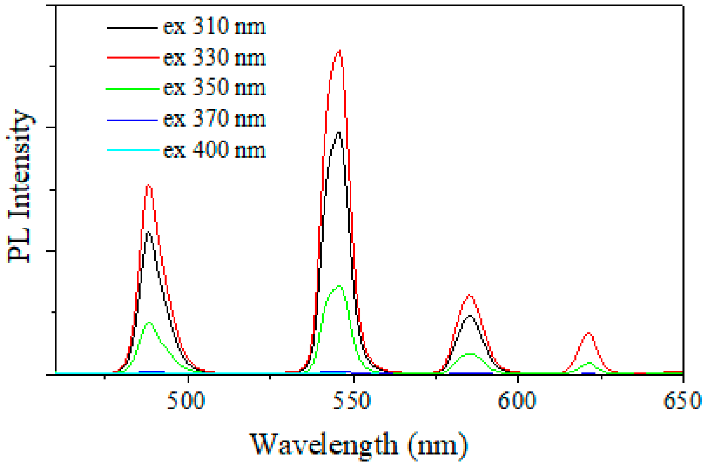

Figure S2. PL spectra of Tb^{III} doped silica nanoparticles at different excitation wavelength

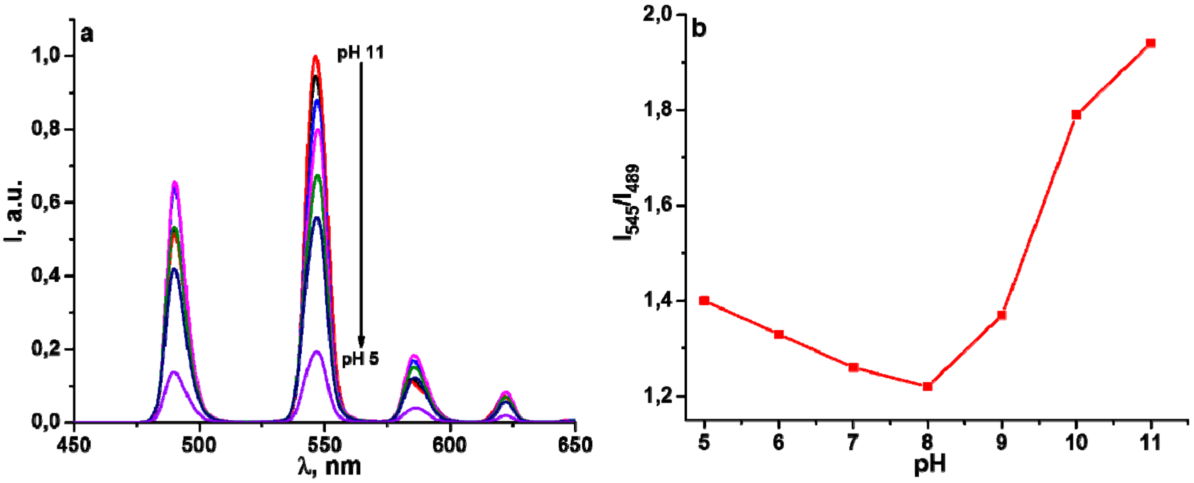

Figure S3. Luminescence spectra (**a**) and ratio of luminescence intensity at 545 and 489 nm (**b**) of Tb^{III}-TCAS complex (0.1 mM) at various pH of aqueous solution (pH was adjusted by NaOH).

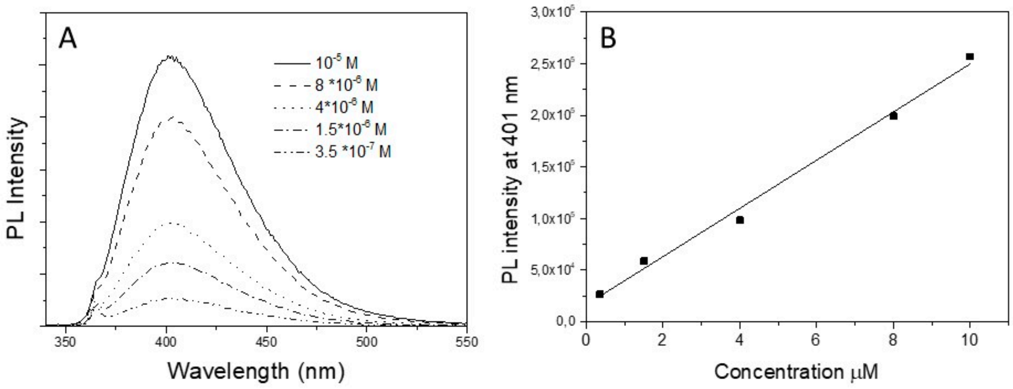

Figure S4. PL spectra (**A**) and calibration curve (**B**) reporting the PL intensity at 401 nm versus the TSPO ligand concentration.
